# Adherence to direct or vitamin K antagonist oral anticoagulants in patients with atrial fibrillation: a long-term observational study

**DOI:** 10.1007/s11239-023-02921-8

**Published:** 2023-12-16

**Authors:** Shahrzad Salmasi, Abdollah Safari, Mary A. De Vera, Tanja Högg, Larry D. Lynd, Mieke Koehoorn, Arden R. Barry, Jason G. Andrade, Marc W. Deyell, Kathy L. Rush, Yinshan Zhao, Peter Loewen

**Affiliations:** 1 Faculty of Pharmaceutical Sciences, University of British Columbia, Vancouver Campus, 2405 Wesbrook Mall, Vancouver, BC V6T 1Z3 Canada; 2 Present Address: Department of Mathematics, Statistics, and Computer Science, University of Tehran, Tehran, Iran; 3 Department of Statistics, University of British Columbia, Vancouver, BC Canada; 4 Centre for Health Evaluation & Outcome Sciences, Providence Health Care Research Institute, Vancouver, BC Canada; 5 School of Population and Public Health, University of British Columbia, Vancouver, BC Canada; 6 Faculty of Medicine, University of British Columbia, Vancouver, BC Canada; 7 UBC Center for Cardiovascular Innovation, Vancouver, BC Canada; 8 School of Nursing, Faculty of Health and Social Development, University of British Columbia Okanagan, Kelowna, BC Canada; 9 Department of Data Analytics, Statistics and Informatics, Faculty of Pharmaceutical Sciences, University of British Columbia, Vancouver, BC Canada

**Keywords:** Atrial fibrillation, Stroke prevention, Anticoagulants, Adherence

## Abstract

Our objectives were to measure long-term adherence to oral anticoagulants (OACs) in patients with atrial fibrillation (AF) and to identify patient factors associated with adherence. Using linked, population-based administrative data from British Columbia, Canada, an incident cohort of adults prescribed OACs for AF was identified. We calculated the proportion of days covered (PDC) as a time-dependent covariate for each 90-day window from OAC initiation until the end of follow-up. Associations between patient attributes and adherence were assessed using generalized mixed effect linear regression models. 30,264 patients were included. Mean PDC was 0.69 (SD 0.28) over a median follow-up of 6.7 years. 54% of patients were non-adherent (PDC < 0.8). After controlling for confounders, factors positively associated with adherence were number of drug class switches, history of stroke or transient ischemic attack, history of vascular disease, time since initiation, and age. Age > 75 years at initiation, polypharmacy (among VKA users only), and receiving DOAC (vs. VKA) were negatively associated with adherence. PDC decreased over time for VKA users and increased for DOAC users. Over half of AF patients studied were, on average, nonadherent to OAC therapy and missed 32% of their doses. Several patient factors were associated with higher or lower adherence, and adherence to VKA declined during therapy while DOAC adherence increased slightly over time. To min im ize the risk stroke, adherence-supporting interventions are needed for all patients with AF, particularly those aged > 75 years, those with prior stroke or vascular disease, VKA users with polypharmacy, and DOAC recipients.

**Supplementary Information:**

The online version contains supplementary material available at 10.1007/s11239-023-02921-8.

## Introduction

Atrial fibrillation (AF), the most common chronic arrhythmia, affects more than 33 million people worldwide [1]. Based on landmark trials showing oral anticoagulant (OAC) therapy [*vitamin K antagonist (VKA)*: warfarin, and *direct oral anticoagulants (DOACs)*: apixaban, dabigatran, edoxaban, rivaroxaban] significantly reduce strokes and deaths, AF practice guidelines recommend people with AF and additional stroke risk factors be prescribed OACs [2]. DOACs are now recommended as first-line therapy for AF stroke prevention, yet VKAs will remain in use for the foreseeable future for AF patients with renal dysfunction, rheumatic valve disease, preference for therapy that can be directly monitored, or for whom the cost of DOACs is prohibitive [2].

Medications cannot exert their full effect if not taken as prescribed. Poor OAC adherence in patients with AF has been associated with significantly higher stroke risk [hazard ratio: 3.6; 95% CI 2.68–5.01] [3]. However, significant gaps in the evidence about AF patients’ adherence to OACs remain [4]. First, although thromboprophylaxis in AF is usually intended to be life-long and adherence is known to decline over time, studies of adherence are mostly limited to the first year of therapy, hence missing numerous aspects of long-term therapy, most notably persistence. Second, despite a high incidence of OAC switches, current evidence is limited to studying the first OAC prescribed to the patient. Third, warfarin has frequently been excluded from studies because its varying daily dose complicates measurement of adherence. As a result, there is a lack of evidence on comparative adherence between VKAs and DOACs. Our objectives were to measure long-term adherence to all OACs in patients with AF while accounting for switching and nonpersistence, and to identify patient factors associated with adherence.

## Methods

### Study design and setting

This was a retrospective observational study using administrative data from Population Data BC (PopDataBC) [5] covering 1996 to end of 2019 for the entire population of British Columbia (BC), Canada (~ 5 million residents). The data was from the following databases: Medical Services Plan (MSP) [6] containing outpatient visits, the Discharge Abstract Database (DAD) [7] containing hospitalizations, the Consolidation File [8] containing demographics such as sex, place of residence, and registration with the provincial healthcare plan, and the Vital Statistics Database [9] containing date and primary cause of death. Information on all medications dispensed outside of hospital between January 1996 and December 2019 was retrieved from PharmaNet and linked to the other datasets. [10]

### Participants

We created an incident cohort of adult patients ≥ 18 years of age with non-valvular AF. Using the algorithm validated by Navar et al. with a positive predictive value of 95.7% [11] we included individuals who had ≥ 3 recorded visits in MSP or DAD related to AF or atrial flutter, with at least one of the three recorded visits being AF-specific (ICD-9: 427.31; ICD-10: I48; Supplemental Appendix A). At least two of the visits had to occur within one year.

**Fig. 1:**
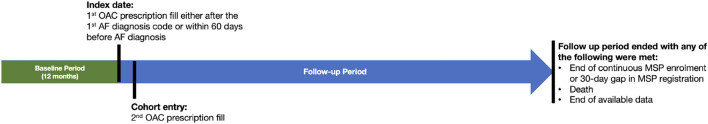
Overview of the study design

Next, using ATC codes from PharmaNet records, we captured all prescriptions for OACs available in the study jurisdiction (warfarin, dabigatran, apixaban, rivaroxaban, edoxaban). Individuals were excluded if they did not have continuous public medical insurance coverage during the 1-year prior to their first OAC prescription fill, or if their first OAC prescription occurred before 1997 to permit 1-year baseline covariate ascertainment, or after Jan 2019 to ensure ≥ 1 year follow-up before end of the data. The date on which the first OAC prescription was filled after the first AF diagnostic code (or within 60 days before the first AF code), was referred to as the “index date” (Fig. 1).

To ensure incident AF cases and incident OAC use, those with an AF code or OAC prescription fill during the 3 years prior to their AF diagnosis date or their index date, respectively, were excluded. To improve specificity, we then excluded individuals with indications for OAC other than non-valvular AF (e.g. venous thromboembolism, rheumatic valve disease; Supplemental Appendix A) based on codes appearing any time before their index date. We also excluded patients with two different OACs filled on the same index date. Finally, because ≥ 2 OAC prescription fills were required to measure adherence, patients with only one OAC prescription fill were excludedPatients were followed from the index date until the end of their follow-up time, defined as December 2019, date of death, or discontinuation of their public medical insurance enrolment, whichever came first.

### Variables

Patient characteristics were measured during the 1-year prior to the index date (the “baseline period”).

To quantify days supply of OAC, the number of days of medication dispensed to the patient was calculated for every prescription fill for every patient (Supplemental Appendix D). For patients on DOACs, whose dosing regimens are relatively fixed, daily dose and days supply was obtained directly from PharmaNet. For warfarin patients whose daily dose changes frequently in response to their International Normalized Ratio (INR) values, the Random Effects Warfarin Days’ Supply (REWarDS) method was used to estimate patient’s daily dose and days supply for Eq. 1.[12] 

**Fig. 2:**
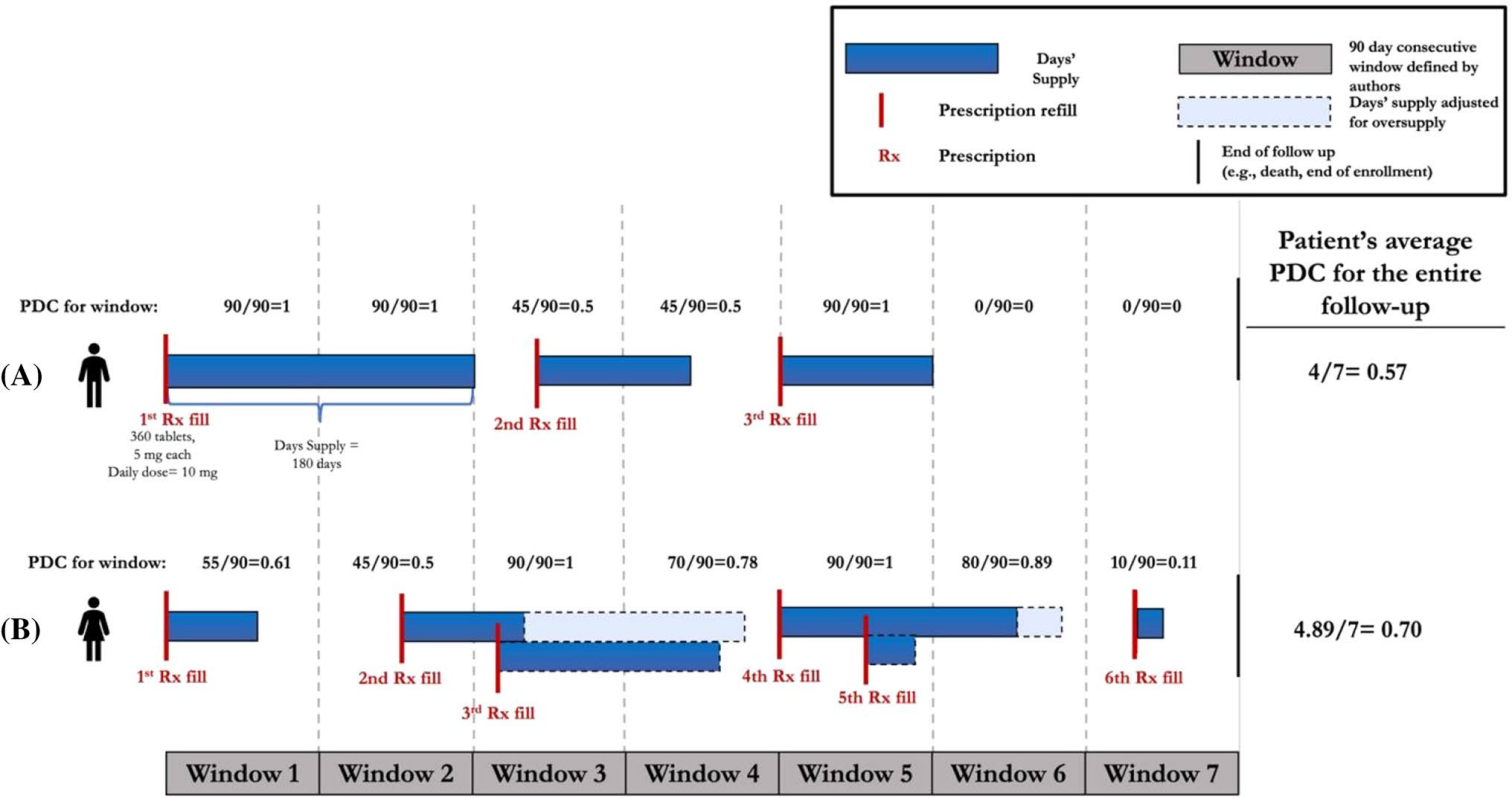
Visual representation of PDC calculation using consecutive windows of 90 days throughout the follow-up period. Scenario **A**: The end of the blue bars denotes the times at which the patients’ supply is expected to run out. The PDC for each window is calculated by dividing the days’ supply in each window by 90 days (the length of the window). Scenario **B**: Oversupply has occurred in windows 3 and 5. The dates of the prescription fills have been adjusted to when the medication supply for the previous refill is estimated to run out

In order to study the execution and persistence phases of adherence, 90-day consecutive time windows (the standard length of dispensing in the study jurisdiction) were created for each patient starting from index date until the end of their follow-up time (Fig. 2, Scenario A). The primary measure of adherence for this study was proportion days covered (PDC). PDC for each 90-day consecutive window was calculated using standard approaches [13]. If a patient permanently discontinued their medication, all consecutive windows after their last supply ran out were assigned PDCs of zero until the end of their follow-up period (Fig. 2A). This approach was based on clinical guideline recommendations for life-long OAC therapy for AF patients (even after experiencing a bleed) [2]. In cases of oversupply, the date of refill for the second prescription was adjusted to when the medication supply for the previous refill was estimated to run out (Fig. 2B). Patient’s average adherence over follow-up was obtained by calculating the mean of PDC values of all their follow-up windows. The mean PDC was reported for the cohort over the entire follow-up. PDC was also analyzed dichotomously with nonadherence defined as PDC < 0.8 and sensitivity analyses conducted with thresholds of 0.7 and 0.9.

Since PharmaNet does not contain information on medications administered during hospitalizations, for our primary analysis, hospitalization periods were removed from both the denominator and numerator when calculating PDC. A sensitivity analysis was conducted with patients assumed to be 100% adherent during periods of hospitalization (Supplemental Appendix C). The Intraclass Correlation Coefficient (ICC) was used to compare the PDCs calculated by the two methods.

### Statistical analysis

Generalized mixed effect linear regression models were used to identify factors associated with adherence. This was limited to the DOAC era, (after October 2010, when the first DOAC was marketed in Canada). Given the repeated data structure (multiple PDC measurements per person), the generalized estimating equation (GEE) technique, with PDC as the response variable, was used to account for the dependencies among data. Drug class (VKA or DOAC) was treated as a time-varying variable to allow for switches during follow-up and examination of patients’ current OAC. Out-of-pocket cost was also a time-varying variable. Other variables were fixed in time. Follow-up time was added to the regression model as an offset term to account for highly variable follow-up times among patients. The “*exchangeable”* correlation structure was found to best fit our data.

Candidate covariates were sex, age, years since OAC initiation, individual components of the CHA_2_DS_2_-VASc [14] stroke and modified HAS-BLED bleeding risk scores [15], weighted Charlson comorbidity index [16], number of concomitant medications (with polypharmacy defined as ≥ 5 concurrent medications, not including OAC on index date) [17], neighbourhood income quintile, out of pocket cost per day supply, and number of drug class switches during follow-up. Time was measured as number of years since the index date. After controlling for confounders, potential interactions between drug class and time, number of drug class switches, history of a major bleed, history of stroke, neighbourhood income quintile, and out-of-pocket prescription cost incurred by patient were evaluated. Statistically significant interaction terms were included in the model. Selection of the initial list of candidate covariates was guided by availability in the data, previous literature [4] and our clinical experience. We used variable selection analysis during the modelling step to develop the final list. To avoid the limitations of traditional variable selection methods such as stepwise (forward or backward) algorithms or selection based on p-values, we used a mix of variable inflation factor (VIF) to avoid collinearity/multicollinearity among the covariates and quasi-likelihood criterion (QIC) to select the best set of covariates with low VIF.

Secular trends in adherence were also analyzed and stratified by OAC drug class.

Analyses were done in R 4.0.3 (R Foundation for Statistical Computing, Vienna, Austria). Database access was approved by the data stewards and the study protocol was approved by the University of British Columbia Clinical Research Ethics Board (approval number: H17-02420).

## Results

The study cohort consisted of 30,264 patients with AF with a median follow-up time of 6.7 (interquartile range [IQR] 6.8) years (8.8 years (IQR 7.5) for index drug VKA; 4.5 years (IQR 3.0) for index drug DOAC). Cohort characteristics are shown in Table 1. The study flow diagram is in Supplemental Appendix B. Our AF cohort included 69,810 people (~ 1% of the BC population), which aligns with 2020 AF prevalence estimates for Canada. [2]

**Table 1:** Characteristics of the cohort

Patient Characteristics	Total 30,264	Female 13,493	Male 16,771
Neighbourhood income quintile, mean (SD)^a,d^	3 (1.42)	2.99 (1.41)*	3.17 (1.42)*
Age at index date, mean (SD)	72.17 (10.96)	75.06 (9.77)*	69.86 (11.32)*
CHA_2_DS_2_-VASc score, median (IQR)^a,b^	3 (1)	3 (2)*	3 (2)*
HAS-BLED, median (IQR)^a,b^	2 (1)	2 (1)*	2 (1)*
Weighted Charlson Comorbidity Index, median (IQR)^a,c^	5 (5)	5 (5)	5 (5)*
Number of concomitant medications, median (IQR)^e^	2 (3)	3 (4)*	2 (4)*
Number of OAC prescription refills over follow-up, median (IQR)	31 (43)	35 (50)*	29 (39)*

*Level of significance for Females vs. Males < 0.001 using unpaired T-test or Wilcoxon rank sum test with continuity correction

*SD* standard deviation; *IQR* interquartile range

^a^Measured during the baseline period

^b^Comorbidities were considered present if at least one disease-specific ICD code was present in either outpatient or inpatient records, during the baseline period. Maximum possible score is 9

^c^Score range: 0–31. Zero indicates no comorbidities were found

^d^The average equivalized disposable income by postal code in patients’ residential area at index date, with 1 as the lowest and 5 as the highest level

^e^Number of medications used continuously (with no gap) for at least three months in the 6-months prior to index date

The average PDC for the cohort was 0.68 (SD 0.28). This means that patients missed, on average, 32% of their doses. Using the conventional threshold for adherence of 0.8, 16,183 (54%) of the patients were classified as non-adherent.

**Fig. 3:**
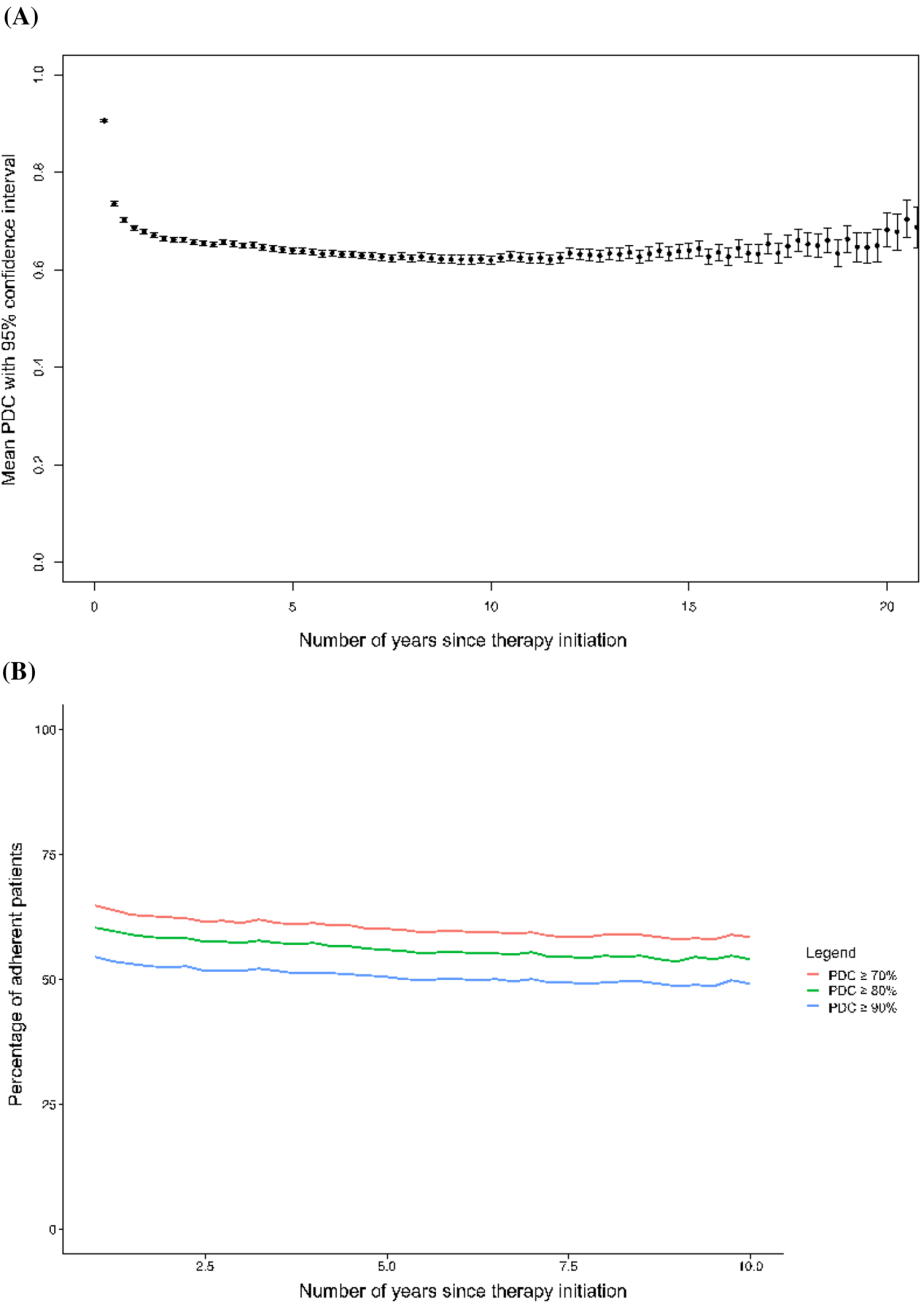
**A** Mean PDC over time with 95% confidence intervals. **B** Proportion of adherent patients over time using different PDC adherence thresholds

Figure 3 depicts adherence over time. Unadjusted analyses of current OAC showed OAC-specific overall mean PDCs of 0.56 (SD 0.45, n = 22,253) for VKA (i.e., warfarin), 0.74 (SD 0.40, n = 17,488) for DOACs overall, 0.77 (SD 0.37, n = 6,786) for apixaban, 0.60 (SD 0.46, n = 5496) for dabigatran, 0.67 (SD 0.42, n = 78) for edoxaban, and 0.70 (SD 0.42, n = 9460) for rivaroxaban.

Factors significantly and positively associated with adherence were time since OAC initiation (0.8% increase per year; estimate 1.008, 95%CI 1.005–1.011), number of drug class switches (3.5% increase in PDC; estimate: 1.035, 95%CI 1.031–1.038), history of stroke or transient ischemic attack (6.7% increase in PDC, estimate: 1.067, 95%CI 1.032–1.103), history of vascular disease (2.8% increase in PDC, estimate: 1.028, 95% CI: 1.005–1.052), and age (0.3% increase in PDC for every 5 year increase in age, estimate: 1.003, 95%CI 1.003–1,003). Age > 75 years when initiating OAC was associated with 12% lower PDC (estimate 0.885; 95%CI 0.856–0.915). Out of pocket cost paid by patient and neighbourhood income quintile were not significantly associated with adherence despite significant differences in costs of the drugs in Canada. Regression model results are in the Supplemental Appendices.

After controlling for confounders, statistically significant interactions were identified between drug class and time since OAC initiation, as well as drug class and the number of concomitant medications. For DOAC recipients, addition of each concomitant medication was associated with a 1% increase in adherence (estimate: 1.01, 95%CI 1.00–1.01), while for VKA recipients each additional concomitant medication was associated with a 4% decrease in PDC (estimate: 0.96, 95%CI 0.941, 0.969).

Regarding secular trends, PDC increased by 1% per year for DOAC recipients (estimate: 1.01, 95%CI 1.01–1.01), but decreased 12.5% per year for VKA recipients (estimate: 0.875, 95%CI 0.865–0.886). Addition of one medication to patients’ concomitant medications was associated with a 1% increase in PDC (estimate: 1.01, 95%CI 1.00–1.01) for DOAC and 4% decrease in PDC for VKA (estimate: 0.96, 95%CI 0.941–0.969).

Being on VKA was associated with 13% higher PDC compared to being on DOAC, after controlling for all confounders and taking into account the interactions (estimate: 1.13, 95%CI 1.11–1.15).

The two approaches to accounting for hospitalization produced similar results: mean PDC with hospitalization excluded was 0.685 (SD 0.279) and was 0.687 (0.278)] with hospitalization included [ICC 0.998 (95% CI 0.998–0.998)].

## Discussion

Over half the patients in our cohort were nonadherent to their OAC, missing, on average, 32% of their doses. The PDC obtained in our study was lower than the pooled mean PDC reported in a recent meta-analysis [PDC one-year post therapy initiation: 0.74 (95% CI: 0.68, 0.80)] [4]. This was expected since our study included the execution and persistence phases of adherence, the latter of which is usually overlooked, as is warfarin use, which we were able to include [4]. Further, our median follow-up time was 6.8 years, much longer than other studies which were mostly confined to the first year of therapy. Also unique to our study was the use of 90-day consecutive windows and repeated measure regression analysis which allowed us to study current drug class as a time-varying variable despite the presence of switching. The observed overall declining pattern of adherence in the first year after therapy initiation is similar to that reported for statins, antihypertensives, and antiplatelets and emphasizes the need for interventions to support adherence at the outset of therapy [18]. We also found that the two methods commonly used in the literature to address hospitalization had no differential impact on adherence estimates.

More frequent drug class switches was associated with higher adherence. This may reflect iterative increases in patient satisfaction with therapy as it was changed, but exploration of this phenomenon, including the reasons for switching and its impact on clinical outcomes is warranted for future studies. The positive associations we found between previous stroke and vascular disease with adherence may be due to heightened awareness of consequences of non-adherence to OACs in these patients. Our finding of a clinically trivial relationship between polypharmacy and adherence for DOACs but a much larger negative effect of polypharmacy on VKA adherence may be attributed to the additional burdens of VKA (lab testing, dietary restrictions, drug interactions) compared to DOACs, which might increase disproportionally in polypharmacy. We are the first to identify an interaction between OAC drug class and number of concomitant medications. The inconsistent findings in the literature on the impact of concomitant medications on adherence may be due to lack of accounting for this interaction [4].

Increasing age at time of OAC initiation was associated with better adherence, but being older than 75 years was associated with lower adherence. Our study is among the largest and longest-term studies of OAC adherence and provides the clearest picture to date of a possible peak in adherence in the 8th decade of life, with waning adherence thereafter for any number of reasons, possibly including placing declining value on prevention, accumulation of comorbidities, risk of adverse events (e.g. bleeding), cognitive decline, attitudes of care providers toward preventive therapies, and deprescribing. This peak effect may be the source of inconsistent findings on the impact of age on adherence in AF in the literature [4]. Interestingly, out-of-pocket cost and patients’ income quintile had no impact on adherence despite the marked difference in cost between VKA and DOAC in Canada. This could be because of the high baseline income quintile of our cohort (3.3 on a scale of 5), or because patients who couldn’t afford DOACs remained on VKA.

The higher overall adherence to VKA than DOAC we observed may be attributable to VKA patients’ higher awareness of therapy because regular contact with the healthcare system for INR testing and the dietary implications of warfarin. On the other hand, adherence declined over time for VKA while it increased for DOACs, possibly reflecting more fatigue with VKA’s monitoring inconveniences. Shore et al. showed that every 10% decline in PDC is associated with a 13% increase in risk of combined all-cause mortality and stroke (aHR:1.13, 95% CI 1.07–1.19) but was not associated with significant change in risk of non-fatal bleeding events (aHR:1.04, 95% CI 0.94–1.14) [19]. Collectively, these findings indicate that prescribers should not assume inherently better adherence for DOACs and should choose OACs through shared decision-making processes that include patient education and clarification of patients’ values and preferences as much as possible [2]. They also point to the need for better adherence-enhancing interventions for all AF patients prescribed OACs, particularly early in the course of therapy.

The main strength of the current study is the data we used British Columbia’s population database which, unlike most, has longitudinal data on all prescription fills regardless of who pays, making it excellent for drug exposure studies [5]. The limitations of this study include those inherent to the use of administrative data. We assumed that filling a prescription equates to consuming the medication, which may not always be true. Some variables known to affect adherence [e.g. education level, frailty, nonprescription drug use (e.g. NSAIDs, ASA), adverse effects experienced, psychosocial variables] were absent from the databases, limiting our ability to account for their contributions [20]. Relatively frequent switching between OACs hampered robust comparisons between individual agents or once vs. twice daily-dosed OACs. Further, we were unable to distinguish clinician-guided therapy discontinuations (temporary or permanent) from patient-initiated ones in our calculation of PDC. Considering the life-long nature of thromboprophylaxis in AF patients and the few absolute contraindications to OACs, we believe this to have had a minor impact in our estimation of OAC adherence. Further, requiring at least two prescription refills for inclusion in the cohort means that we excluded the most non-adherent patients (i.e., those who never initiated therapy or stopped after one prescription fill), hence possibly overestimating adherence. Finally, the data sources did not allow reliable classification of long-term care status of patients, a group with potentially higher adherence. Despite this, the current population of > 65 year olds in BC is < 3% and our sensitivity analyses showed PDC was insensitive to methods of accounting for inpatient days, so the probability that our results were affected by our not classifying long-term care residents is low.

This long-term cohort study showed that 54% of patients with AF in British Columbia were nonadherent to their OAC therapy and missed 32% of their doses. Several patient factors were associated with higher or lower adherence, and adherence to VKA declined during therapy while DOAC adherence increased slightly over time. Adherence-supporting interventions are needed for all patients with AF, particularly those aged > 75 years, and those with prior stroke or vascular disease, VKA users with polypharmacy, and DOAC recipients.

### Supplementary Information

Below is the link to the electronic supplementary material.

Supplementary file1 (DOCX 422 KB)

## Data Availability

Access to data provided by the Data Steward(s) is subject to approval, but can be requested for research projects through the Data Steward(s) or their designated service providers. All inferences, opinions, and conclusions drawn in this publication are those of the author(s), and do not reflect the opinions or policies of the Data Steward(s).
