# Supplementary material for: Adherence to direct or vitamin K antagonist oral anticoagulants in patients with atrial fibrillation: a long-term observational study
Source: J Thromb Thrombolysis. 2023 Dec 16;57(3):437–44. doi: 10.1007/s11239-023-02921-8 (PMC10961264; doi:10.1007/s11239-023-02921-8)
Supplement: Supplementary file 1 — Supplementary file1 (DOCX 422 KB) [file 11239_2023_2921_MOESM1_ESM.docx]

# Supplemental Appendices

## ICD codes used

|  | ICD-9 | ICD-10 |
| --- | --- | --- |
| Qualifying AF ICD codes |  |  |
| Cardiac Dysrhythmia | 427 |  |
| Atrial Fibrillation and Flutter | 427.3 | I48 |
| Atrial Fibrillation | 427.31 | I48.0-2, I48.9 |
| Atrial Flutter | 427.32 | I48.3, I48.4 |
| Excluding AF ICD codes |  |  |
| Pulmonary embolism | 415.1 | I26 |
| Phlebitis and thrombophlebitis | 451 | I80 |
| Other venous embolism and thrombosis | 453 |  |
| Disease of mitral and aortic valve | 394,395,395 | I05, I08 |
| Other rheumatic heart diseases | 398 | I06, I09 |
| Budd-Chiari syndrome |  | I82 |
| Exposure for stroke risk calculation: CHA_2_DS_2_-VASc | | |
| **C**ongestive Heart failure | 398.91, 402.01, 402.11, 402.91, 404.01, 404.03, 404.11, 404.13, 404.91, 404.93, 428.x | I50, I11.0, I13.0, I13.2, I42.0 |
| **H**ypertension | 401.x, 402.x, 403.x, 404.x, 405.x, 437.2 | I10-I13, I15 |
| **A**ge¶ | N/A | N/A |
| **D**iabetes | 250.x, 357.2, 362.0, 366.41 | E10, E11, E13, E14 |
| **S**troke/Transient Ischemic Attack | 433.xx, 434.xx, 435.x, 431, 436, 438.x | I63, I64, G45, I69, I74 |
| **Va**scular disease | 410.x, 411.x, 412.x, 413.x, 414.x, 440.x, 447.1, 557.1, 557.9 | I21, I23, I252, I70–73 |
| **S**ex **c**ategory | N/A | N/A |
| Exposure for bleeding risk calculation: Modified† HAS-BLED | | |
| **H**ypertension‡ | 401, 402, 403, 404, 405 | I10-I13, I15 |
| **A**bnormal renal function  (Kidney disease) | 403.01, 403.11, 403.91, 404.02, 404.03, 404.12, 404.13, 404.92, 404.93, 582, 583.0, 583.1, 583.2, 583.3, 583.4, 583.5, 583.6, 583.7, 585, 586, 588.0, V42.0, V45.1, V56 | I12, I13, N00, N01, N02, N03, N04 N05, N07, N11, N14, N17, N18, N19, Q61 |
| **A**bnormal liver function  (Liver disease) | 070.22, 070.23, 070.32, 070.33, 070.44, 070.54, 070.6, 070.9, 456.0, 456.1, 456.2, 570, 571, 572.2, 572.3, 572.4, 572.5, 572.6, 572.7, 572.8, 573.3, 573.4, 573.8, 573.9, V42.7 |  |
| **S**troke or Transient Ischemic Attack | 433.xx, 434.xx, 435.x, 431, 436, 438.x | I63, I64, G45, I69, I74 |
| **B**leeding history (Major) | 280.0, 285.1, 423.0, 430, 431, 432.x, 455.2, 455.5, 455.8, 459.0, 456.0, 456.20, 459.0, 530.21, 530.7, 530.82, 531.0x, 531.2x, 531.4x, 531.6x, 532.0x, 532.2x, 532.4x, 532.6x, 533.0x, 533.2x, 533.4x, 533.6x, 534.0x, 534.2x, 534.4x, 534.6x, 535.01, 535.11, 535.21, 535.31, 535.41, 535.51, 535.61, 535.71, 537.83, 537.84, 562.02, 562.03, 562.12, 562.13, 568.81, 569.3, 569.85, 578.x  852.x, 853.x  596.7, 599.7, 719.1x, 784.7, 784.8, 786.3 | I60, I61, I62, K250, K252, K254, K260 K262, K264, K270, K272, K274, K280, K282, K290, K920, K921, K922, D62, J942, H113, H356, H431, N02, R04, R31, R58 |
| **L**abile INR (NA – modified HAS-BLED) | N/A | N/A |
| **E**lderly >65 years old ¶ | N/A | N/A |
| **D**rug or  Alcohol use  (Alcoholism)§ | 265.2, 291.1, 291.2, 291.3, 291.5, 291.6, 291.7, 291.8, 291.9, 303.0, 303.9, 305.0, 357.5, 425.5, 535.3, 571.0, 571.1, 571.2, 571.3, 980, V11.3 | E224, E529A, F10, G312, G621, G721 I426, K292, K70, K860, L278A, O354 T51, Z714, Z721 |
| † Modified to omit labile INR, which isn’t ascertainable from Population Data British Columbia.  ¶ Calculated at time of first OAC prescription  ‡ To overcome the under-coding of hypertension in Population Data British Columbia we utilized alternative coding to detect hypertension cases, a method used previously by our team members in this particular database. We identified subjects with hypertension from combination treatment with at least two classes of antihypertensive drugs.  § Refers to use of Non-Steroidal anti-inflammatory drugs (NSAID) and/or acetylsalicyclic acid. The ATC codes for these medications can be found in table A4.  ICD: International Classification of diseases  ATC: Anatomical Therapeutic Chemical | | |

Table A1: International Classification of Diseases (ICD) codes used in identifying AF cases and calculating patient’s bleeding and stroke risk scores.

| Drug class | Anatomical Therapeutic Chemical (ATC) code |
| --- | --- |
| Antihypertensive medications used to assist in ascertaining the diagnosis of hypertensions. | |
| Alpha adrenergic blockers | C02A, C02B, C02C |
| Non-loop diuretics | C02DA, C02L, C03A, C03B, C03D, C03E, C03X, C07C, C07D, C08G, C09BA, C09DA, C09XA52 |
| Vasodilators | C02DB, C02DD, C02DG, C04, C05 |
| Beta blockers | C07 |
| Calcium channel blockers | C07F, C08, C09BB, C09DB |
| Renin-angiotensin system inhibitors | C09 |
| Medications used in calculation of the HASBLED score. | |
| Platelet aggregation inhibitors excluding heparin | |
| acetysalicyclic acid | B01AC06 |
| clopidogrel | B01AC04 |
| prasugrel | B01AC22 |
| ticagrelor | B01AC24 |
| Anti-inflammatory and antirheumatic products, non-steroids | |
| oxicams, acetic acid derivatives, Butylpyrazolidines, Propionic acid derivatives, Fenamates, Coxibs | M01A |

Table A2: ATC codes for medications used in calculation of the HASBLED score.

## Study flow chart


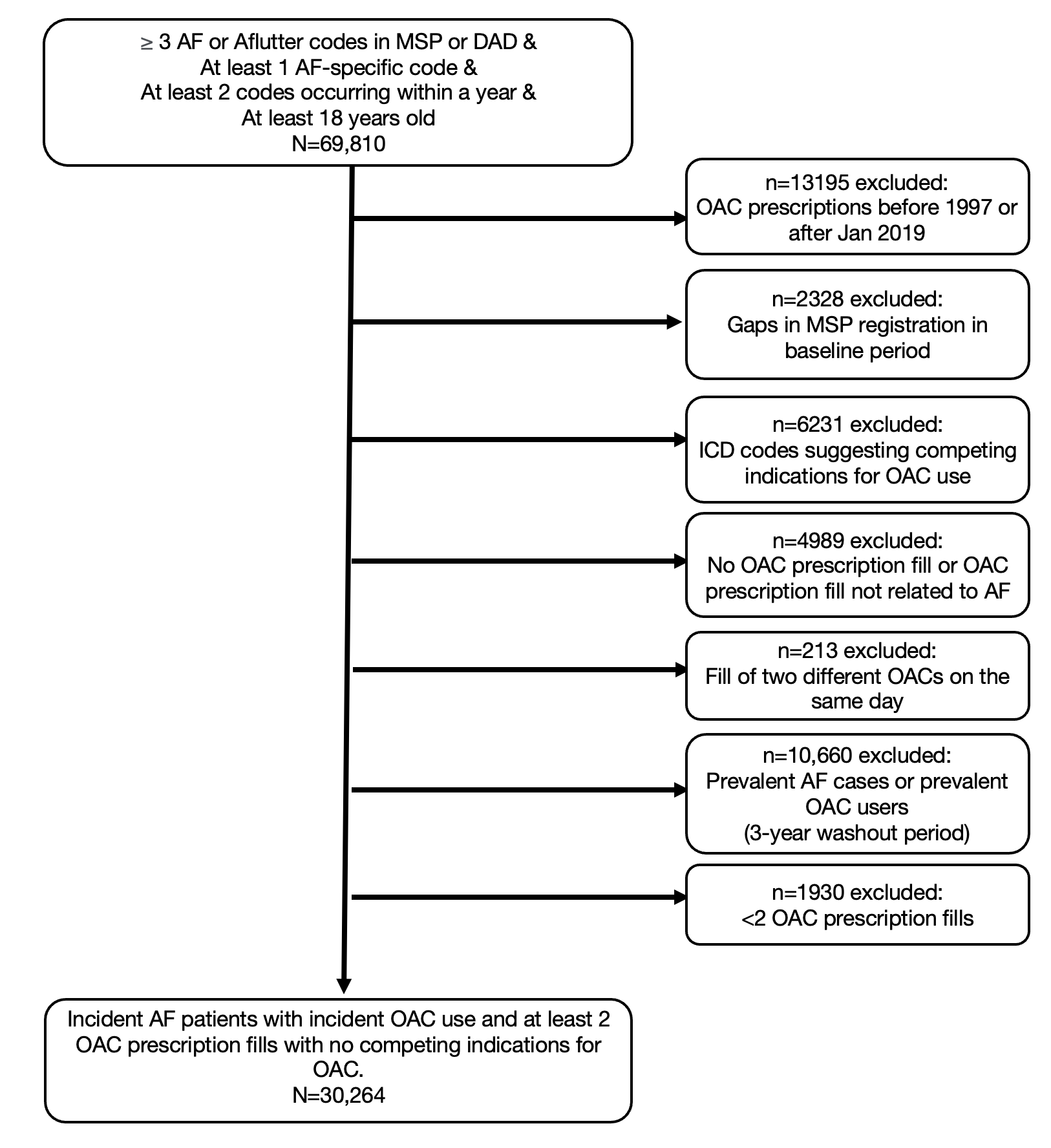


## Addressing hospitalization periods when calculating PDC


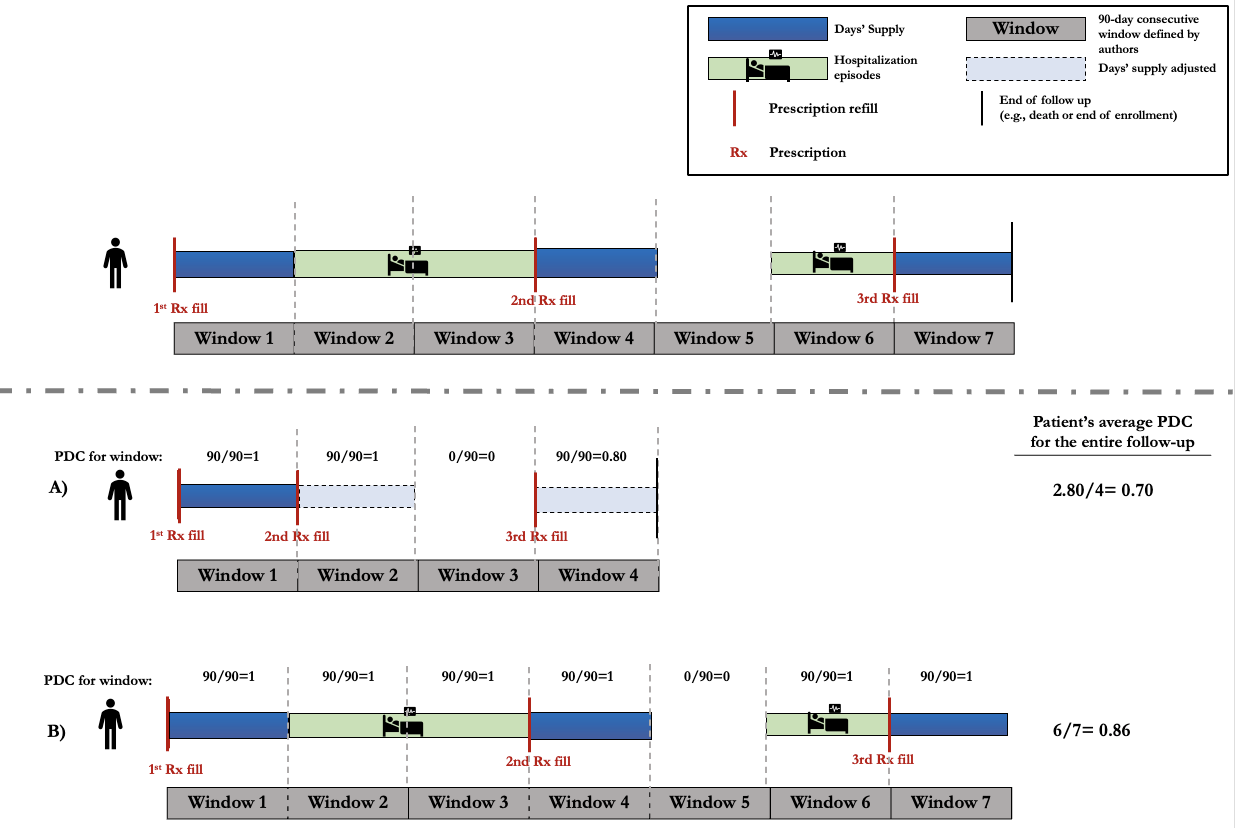


Figure C1: Visual representation of addressing hospitalization periods when calculating PDC using consecutive windows of 90 days throughout the follow-up period. Scenario A: Hospitalization episodes have been removed from the numerator (number of days covered) and denominator (follow-up time) in the PDC calculation. Scenario B: Hospitalization has been included in the PDC calculation, patients were assumed to have been 100% adherent during these periods.

## Days’ Supply Calculation

Days supply_ij_ = (number of tablets dispensed_ij_ × tablet strength_ij_) / patient’s daily dose in milligrams_i._

Where j = every prescription fill and refill, i = every patient, n = number of patients, and m_i_ = number of prescription fill for the patient

To quantify days supply of OAC, the number of days of medication dispensed to the patient was calculated for every prescription fill and refill j, j = 1, …, m_i_, and for every patient i, i = 1, ... n

## Regression analysis of factors associated with OAC adherence during the DOAC era (n= 15,949)

| Outcome:  Continuous PDC^††^ | Effect estimate^†^ | 95% Confidence Interval^†^ |
| --- | --- | --- |
| Time (Number of years since OAC initiation) ^a^ | 1.008 | 1.005, 1.011 *** |
| Number of concomitant medications ^e^ | 1.008 | 1.004, 1.013 *** |
| $CAD paid out of pocket per day supply ($100 dollar intervals) ^a^ | 1.000 | 1.000, 1.000 |
| Weighted Charlson Comorbidity Index ^b^ | 0.998 | 0.989, 1.007 |
| Age (5-year intervals) ^e^ | 1.003 | 1.003, 1.003 *** |
| Number of drug class switches over follow-up ^a^ | 1.035 | 1.031, 1.038 *** |
| History of major bleeding ^b^ | 0.982 | 0.940, 1.025 |
| History of stroke/TIA ^b^ | 1.067 | 1.032, 1.103 *** |
| Male sex (reference: female) | 0.989 | 0.969, 1.010 |
| Neighborhood income quintile ^c^ | 1.001 | 0.994, 1.009 |
| Diabetes ^b^ | 1.018 | 0.993, 1.044 |
| Hypertension ^b^ | 1.070 | 1.024, 1.119 ** |
| Heart Failure ^b^ | 0.999 | 0.970, 1.030 |
| Vascular disease ^b^ | 1.028 | 1.005, 1.052 * |
| >75 years old at index date ^b^ | 0.885 | 0.856, 0.915 *** |
| Alcohol use ^b^ | 0.922 | 0.835, 1.019 |
| Abnormal liver function ^b^ | 0.916 | 0.787, 1.066 |
| Abnormal renal function ^b^ | 0.972 | 0.929, 1.018 |
| NSAID and ASA use ^b, d^ | 1.002 | 0.999, 1.004 |

ICD-9/10 codes for variables are in Supplemental Appendix A

† Regression model was constructed on a log scale. Effect estimates and the 95% confidence interval have been exponentiated for ease of interpretation. For continuous variables, regression coefficients indicate the percent change in PDC for a one-unit change in the explanatory variable, controlling for all the other variables in the model. For categorical variables, the coefficient indicates the percent change in PDC for specified category versus the reference, controlling for all the other variables in the model.

†† Outcome is continuous PDC for each 90-day window for each patient

^a^ Time-varying variable

^b^ Fixed-in-time variable measured during the baseline period

^c^ The average equivalized disposable income by postal code in patients’ residential area at index date, with 1 as the lowest and 5 as the highest level.

^d^ Not well captured in our database. Our data does not include information about over the counter supply of medications.

^e^ Number of medications used continuously (with no gap) for at least three months in the 6-months period prior to index date.

Level of significance: 0 ‘***’, <0.001 ‘**’, <0.01 ‘*’, <0.05 ‘.’
